# Ionone Is More than a Violet’s Fragrance: A Review

**DOI:** 10.3390/molecules25245822

**Published:** 2020-12-10

**Authors:** Lujain Aloum, Eman Alefishat, Abdu Adem, Georg Petroianu

**Affiliations:** 1 Department of Pharmacology, College of Medicine and Health Sciences, Khalifa University of Science and Technology, Abu Dhabi 127788, UAE; lujain.aloum@ku.ac.ae (L.A.); Eman.alefishat@ku.ac.ae (E.A.); Abdu.Adem@ku.ac.ae (A.A.); 2 Center for Biotechnology, Khalifa University of Science and Technology, Abu Dhabi 127788, UAE

**Keywords:** ionone, biological activity, BCO2, ionone derivatives, cancer, inflammation, OR51E2

## Abstract

The term ionone is derived from “iona” (Greek for violet) which refers to the violet scent and “ketone” due to its structure. Ionones can either be chemically synthesized or endogenously produced via asymmetric cleavage of β-carotene by β-carotene oxygenase 2 (BCO2). We recently proposed a possible metabolic pathway for the conversion of α-and β-pinene into α-and β-ionone. The differences between BCO1 and BCO2 suggest a unique physiological role of BCO2; implying that β-ionone (one of BCO2 products) is involved in a prospective biological function. This review focuses on the effects of ionones and the postulated mechanisms or signaling cascades involved mediating these effects. β-Ionone, whether of an endogenous or exogenous origin possesses a range of pharmacological effects including anticancer, chemopreventive, cancer promoting, melanogenesis, anti-inflammatory and antimicrobial actions. β-Ionone mediates these effects via activation of olfactory receptor (OR51E2) and regulation of the activity or expression of cell cycle regulatory proteins, pro-apoptotic and anti-apoptotic proteins, HMG-CoA reductase and pro-inflammatory mediators. α-Ionone and β-ionone derivatives exhibit anti-inflammatory, antimicrobial and anticancer effects, however the corresponding structure activity relationships are still inconclusive. Overall, data demonstrates that ionone is a promising scaffold for cancer, inflammation and infectious disease research and thus is more than simply a violet’s fragrance.

## 1. Chemical Structure, Physicochemical Properties and Natural Occurrence

Ionone is a ketone compound composed of 13 carbons with a monocyclic terpenoid backbone [1]. The term ionone derived from “iona” (Greek for violet) referring to the violet scent and “ketone” in relation to its structure [2].

Several isomeric ionones exist in Nature, including α-ionone, β-ionone and others. Ionones are found as secondary plant metabolism products that share mevalonic acid as a common precursor [3]. They are widely present in fruits and vegetables comprising β-carotene and are found in plant oils, for example oils from *Petunia hybrida, Boronia megastigma* Nees and especially *Viola odorata* [4,5,6,7]. It can be found in cow’s milk where is passively transferred after consumption of alfalfa pasture [8,9].

α-Ionone and β-ionone are 3-buten-2-ones substituted by 2,6,6-trimethyl-2-cyclohexen-1-yl and 2,6,6-trimethyl-1-cyclohexen-1-yl groups respectively, at position 4 (Table 1) [10,11]. The ionone stereoisomers, α-ionone and β-ionone, are pale yellow-to-yellow liquids [12] with a woody floral scent. However, the former has an extra honey olfactory aspect [13]. α-Ionone and β-ionone share similar physicochemical properties [10,11,12,14,15,16,17,18]. They are both soluble in most fixed oils, alcohol and propylene glycol but insoluble/very slightly soluble in glycerin/water, respectively [12].

α-Ionone and β-ionone are both widely used as aroma ingredients in various industries, including cosmetics (e.g., shampoos, soaps) and non-cosmetic (e.g., household detergents and cleaners) products. The Flavor and Extract Manufacturer’s Association (FEMA) declares that both ionones are Generally Recognized As Safe (GRAS) when used as flavoring agents. The US FDA has also approved their use as flavoring agents [10,11].

## 2. Synthesis

### 2.1. Chemical Synthesis

Violet scent (ionone) is currently used in many commercial products as its price is low. However, before the 1890s violet flower oil was considered the most precious of all essential oils. This is because the production of one kilogram of violet oil requires 33,000 kg of violet flowers [19], which cost approximately 82,500 German gold marks for raw materials. This costly procedure motivated many chemists and industries to find a cost-efficient way to synthesize this oil [20].

**Scheme 1:**
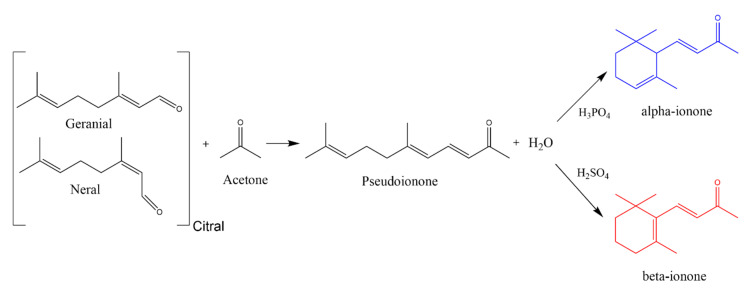
The chemical synthesis of ionones via the Tiemann-Krueger synthesis. Citral and acetone are condensed in an alkaline medium to yield pseudoionone. Pseudoionone is cyclized to ionone upon exposure to acidic conditions. Phosphoric, fumaric and other weaker acids mainly yield α-ionone, while concentrated sulfuric acid preferentially yields β-ionone.

Thus in 1893, Tiemann and Krueger investigated the compound responsible for the scent of violets by studying orris root oil; a much cheaper oil with a similar odour principle [20,21]. They achieved the synthesis of ionone in two steps. First, they performed an aldol condensation of citral with acetone (dimethyl ketone) in an alkaline medium to produce pseudoionone. Then pseudoionone was cyclized to ionone upon exposure to acidic conditions (Scheme 1). The type and concentration of acid used determines the ratio of α-ionone and β-ionone produced in this reaction. For example, phosphoric, fumaric and other weaker acids mainly yield α-ionone while concentrated sulfuric acid preferentially yields β-ionone [22,23]. This synthesis marked the start of fragrance chemistry (Duftstoff-Chemie) [2]. Today the global usage of α-ionone and β-ionone is approximately between 100 and 1000 metric tons yearly [10,11].

### 2.2. Endogenous Synthesis

#### 2.2.1. β-Carotene Oxygenase 1 (BCO1)

**Scheme 2:**
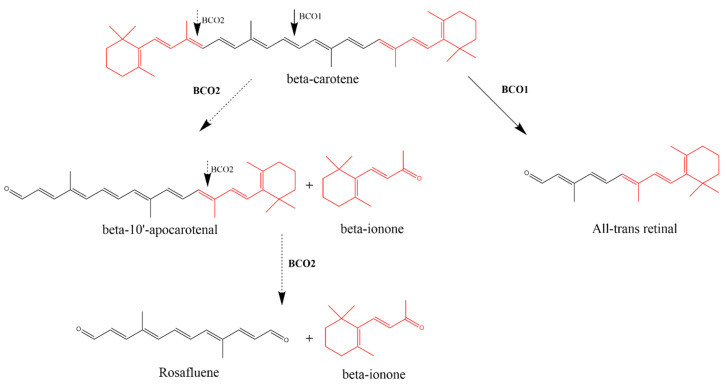
The enzymatic cleavage of β-carotene by β-carotene oxygenase 1 (BCO1) and β-carotene oxygenase 2 (BCO2). The central cleavage of β-carotene catalyzed by BCO1 at the 15,15′ double bond results in the formation of two molecules of all-*trans* retinal. The asymmetric cleavage of β-carotene at the 9,10 double bond facilitated by BCO2 results in generation of β-ionone and β-10′-apocarotenal. Another cleavage at the 9′,10′ double bond yields an additional β-ionone moiety and rosafluene.

Carotenoids are naturally occurring compounds found in fruits and vegetables. They are isoprenoids with a characteristic long conjugated double-bond chain in their structures [24]. In humans, the main carotenoids naturally found are α-carotene, β-carotene, β-cryptoxanthin, lycopene and lutein [25]. The central oxidative cleavage of β-carotene at the 15,15′ double bond results in two all-*trans* retinal entities (Scheme 2). The all-*trans* retinal can then either be oxidized to all-*trans*-retinoic acid or reduced to all-*trans*-retinol (vitamin A), respectively. This process is carried out by the cytosolic enzyme β-carotene oxygenase 1 (BCO1), also identified as β-carotene-15,15′-mono-oxygenase 1 (BCMO1) [24,26]. It was concluded that for the carotenoid to be converted to vitamin A, the carotenoid should contain a minimum of one non-substituted β-ionone ring [27,28], thus BCO1 is only able to facilitate the cleavage of β-cryptoxanthin, α-carotene and β-carotene [28].

#### 2.2.2. β-Carotene Oxygenase 2 (BCO2)-Mediated Synthesis

Vogt and von Lintig, at the University of Freiburg in Germany, first described endogenous ionone synthesis by the asymmetric cleavage of β-carotene mediated by an enzyme. However, von Lintig at Case Western Reserve University in the United States carried further investigations on this enzyme [29,30]. β-Ionone and β-apo-10′-carotenal are the products of asymmetric cleavage of β-carotene (Scheme 2). This cleavage at the 9′,10′ double bond is facilitated by β-carotene oxygenase 2 (BCO2), also known as β-carotene-9′,10′-dioxygenase 2 (BCDO2). Another cleavage at the 9′,10′ double bond yields an additional β-ionone moiety and rosafluene, a compound naturally occurring in roses (Scheme 2) [24,29,31,32].

The difference between the BCO1 and BCO2 enzymes is not confined to their mechanism of cleavage (Table 2). BCO2 can asymmetrically cleave carotenoids that contain hydroxylated ionones and acyclic carotenoids including non-provitamin A carotenoids, for instance lycopene and xanthophylls and thus has broader substrate specificity [31]. Furthermore, BCO2 is localized in the inner mitochondrial membrane [33] while BCO1 is a cytoplasmic protein [28]. These differences between BCO1 and BCO2 suggest unique physiological roles [32].

The central cleavage (via BCO1) of β-carotene represents the primary pathway for retinoid production. It was believed that the asymmetric cleavage (via BCO2) of β-carotene is a salvage pathway for retinoid production [34]. Later it was reported that BCO1-knockout mice with intact BCO2 were vitamin A deficient [35]. In addition, only BCO2 but not BCO1 is expressed in prostate and endometrial connective tissue, endocrine pancreas and cardiac and skeletal muscle. Notably, some of these tissues are not sensitive to vitamin A deficit, thus BCO2 is suggested to play a physiological role independent of vitamin A synthesis [36]; implying that β-ionone (one of BCO2 products) may be involved in other prospective biological functions [37].

BCO2 may play a role in the prevention of hepatic steatosis [32]. Deficiency/mutations in BCO2 led to buildup of carotenoids in adipose tissue in various animals and in mitochondria in a mouse model [32,33,38,39]. Carotenoids in turn can cause oxidative stress and thus apoptosis. Thus, BCO2 was found to keep cells protected from apoptosis brought by carotenoid-induced oxidative stress [40].

The association between the level of β-ionone and BCO2 mutations has not been investigated. It appears likely that mutations or absence of BCO2 would result in reduced levels of β-ionone and might contribute in the pathogenesis of the mentioned diseases. In addition, the discovery of endogenous production of β-ionone indicates that β-ionone might have physiological roles that are yet to be revealed. This is supported by the fact that high intake of fruits and vegetables, which contain volatile isoprenoids such as ionones, is associated with lower risk of cancer [41]. Studies have focused to understand the biological role of apocarotenoids generated by BCO2 [42], β-ionone has been less studied. Thus, in this review, we will present the data describing the physiological effects of ionone.

### 2.3. Possible Ionone Synthesis from Pinene

Pinene is the major constituent of turpentine oil. The turpentine derived from the resin of terbenith tree had been widely used as a medicine and in wine as a preservative agent and taste enhancer. It was noticed that the consumption of turpentine oil alters the urine scent into violet. Anecdotally, turpentine oil was allegedly used by Cleopatra for this purpose. However, mixing turpentine oil with urine doesn’t confer the urine violet-like scent. It was assumed that ionone is the compound accountable for the scent of violet. Thus, it was concluded that pinene (the most prevalent compound in turpentine) should be converted into ionone and then the latter must be renally excreted to produce the violet scent of urine. Hepatic enzymes (cytochrome P450) might be responsible for the conversion of pinene to ionone [2].

**Scheme 3:**
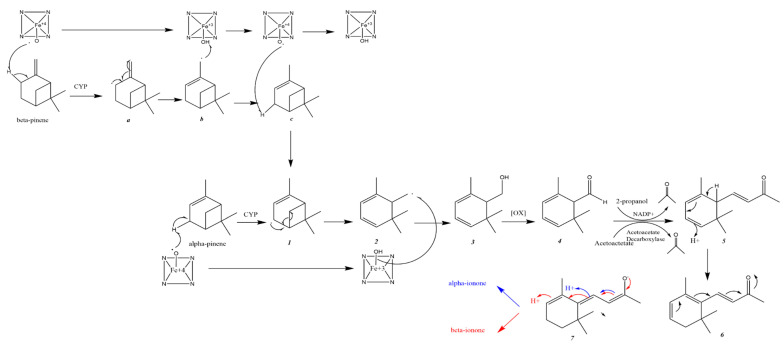
Possible metabolic conversion of α- and β-pinene to α- and β-ionone (modified from [43]).

Al-Tel and his colleagues suggested a possible metabolic pathway for the conversion of α-and β-pinene into α-and β-ionone (Scheme 3). First, the CYP enzyme would oxidize α-and β-pinene generating intermediates (1) and (a), respectively. The intermediate generated from β-pinene (a) will undergo free radical rearrangement forming intermediate (b). The latter will abstract a hydrogen radical from CYP to yield intermediate (c).

This is followed by CYP abstracting the hydrogen radical from intermediate (c). At this point, it is postulated that the two pathways meet via the formation of intermediate **1**. Subsequent free radical rearrangement and cleavage of the cyclobutyl ring should yield intermediate **2**. The latter undergoes oxidation into alcohol **3** and then into aldehyde **4**. Intermediate **4** undergoes a Knoevenagel condensation with acetone to produce **5**. 1,5-Sigmatropic rearrangement of intermediate **5** will yield intermediate **6**. The latter can rearrange to form intermediate **7**, which can later further rearrange into either α- or β-ionone. Acetone is a critical player in this suggested metabolic conversion of pinene to ionone. It could be generated in creatures either via decarboxylation of acetoacetate or dehydrogenation of 2-propanol. The former route constitutes the main source of acetone in mammals [43].

The proposed catalytic reactions were supported by calculated physicochemical properties. The α- and β-ionones had higher hydrophilicty than α- and β-pinenes, which suggests pinene conversion to ionone to facilitate renal excretion. Predicted sites of pinene metabolism via human CYP isoform were reported and CYP isoforms with high probabilities of involvement were: 1A2, 3A4, 2C19 and 2D6 [43].

## 3. Olfactory Receptors

Olfactory receptors (OR) are G-protein coupled receptors that detect volatile substances [44,45]. In olfactory sensory neurons, OR bind to G_olf_ [46] (G protein similar to Gαs) followed by activation of an adenylate cyclase and production of cAMP which would result in an increase in intracellular calcium [47,48,49,50]. In 1992, Parmentier and his colleagues recognized the first OR gene transcript in mammalian germ cells [51]. Further studies displayed that OR expression is not constrained to olfactory epithelium, however they are unexpectedly expressed in all human tissues studied to date such as colon, lung, ovary, liver, kidney, lymph node, heart, blood, testis, skeletal muscle, skin, adipose, adrenal, brain, breast, prostate, and thyroid. Over 40 different OR have been identified, which are found to be expressed in more than 45 different human tissues. This ectopic expression of OR suggests that the function of OR is more than odor detection and discrimination [45,52,53,54]. Studies reported various functions of ectopically expressed OR such as regulation of serotonin release in enterochromaffin cells [55], cancer cell proliferation [56], cytokinesis [57], blood pressure, enzyme secretion in kidneys [58,59] and sperm chemotaxis [60,61,62,63].

Several studies showed that ionone binds to OR51E2 [45,49,56,64,65,66,67,68]. Essentially OR51E2 is identified as ionone receptor belonging to OR family 51 and subfamily E where is it member 2 [30]. OR51E2 is not only one of the most expressed ORs at mRNA level; it is found in a multitude of tissues. OR51E2 is most abundant in prostate tissue (3-∞ FPKM (fragments per kilobase per million) in comparison to other tissues where it is less abundant such as liver (0–0.01 FPKM), brain (0.01–0.1 FPKM), testis (0.1–1 FPKM) and colon (1–3 FPKM) [54].

OR51E2 expression is augmented in prostate cancer [54,69]. Thus, it is also identified as prostate-specific G-protein-coupled receptor (PSGR) [45,54] and considered a marker for prostate tumor [70]. However, Cao et al. reported that PSGR expression was high in prostate intraepithelial neoplasia but decreased as the disease progressed to prostate cancer. In prostate cancer patients, low PSGR expression was found to be correlated with poor overall survival [66]. The number of OR51E2 is higher in human melanoma cells in comparison to melanocytes [49,68]. In addition, another recent study showed that OR51E2 is the most expressed OR transcript in human adult and fetal retinal pigment epithelium. Interestingly, OR51E2 was not only localized in the plasma membrane but also in the cytosol of benign and cancer prostate epithelial cells, retinal pigment epithelium and melanocytes; specifically in the early endosome antigen 1 of melanocytes [37,49,71].

## 4. Ionone Effects

### 4.1. β-Ionone

β-Ionone has been shown to exert a variety of pharmacological effects, including anticancer, chemopreventive, cancer-promoting, melanogenesis, anti-inflammatory and antimicrobial activity. Several mechanisms and intracellular signaling cascades have been reported explaining these effects. β-Ionone activates OR51E2 [37,49,50,56,65,66,67,68,70] and regulates the activity or expression of cell cycle regulatory proteins [3,72,73,74,75,76], pro-apoptotic and anti-apoptotic proteins [3,9,73,76,77,78,79], HMG-CoA reductase [3,74,80,81,82] and pro-inflammatory mediators [73,83].

#### 4.1.1. OR51E2-Mediated Effects

Studies showed that β-ionone inhibits the proliferation [50,56,66,67] and induced invasiveness [65,66,70] of prostate cancer cells via activation of OR51E2, whereas β-ionone mediated-activation of OR51E2 inhibited proliferation and migration of melanocytes and induced melanogenesis [49,68]. Surprisingly, β-ionone-mediated activation of OR51E2 was found to increase the proliferation and metastasis of retinal pigment cells, activating p44/42 and protein kinase B (AKT) proteins [37].

**Figure 1:**
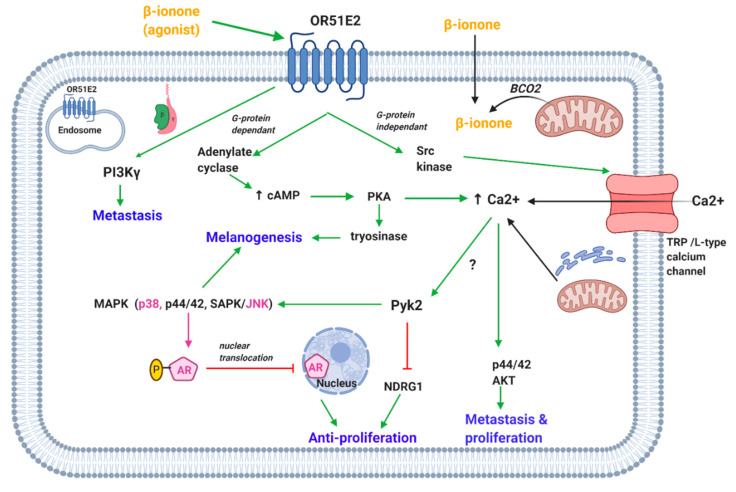
A proposed scheme illustrating the effect of β-ionone mediated activation of membrane and cytosolic OR51E2 receptor leading to an increase in calcium and regulation of various kinases modulating cell proliferation, metastasis and melanogenesis. (OR51E2, olfactory receptor 51E2; BCO2, β-carotene oxygenase 2; cAMP, cyclic adenosine monophosphate; PKA, protein kinase A; TRP calcium channel, transient receptor potential calcium channel; Pyk2, proline-rich tyrosine kinase 2; MAPK, mitogen-activated protein kinase; SAPK/JNK, stress-activated protein kinases/Jun amino-terminal kinases; NDRG1, N-myc downstream regulated gene 1; AR, androgen receptor; AKT, protein kinase B; PI3Kɣ, phosphoinositide 3-kinase gamma).

β-Ionone activation of OR51E2 resulted in modulation of kinases activity and increase in intracellular calcium (Figure 1). The Neuhaus team showed that β-ionone activated OR51E2 (not androgen receptors (AR)) causing an increase in intracellular calcium concentration in both primary prostate cancer epithelial cells and prostate cancer cell line (LNCaP). Further testing elucidated that β-ionone exert the anti-proliferative effect via phosphorylating p38 and stress-activated protein kinase/Jun amino-terminal kinase (SAPK/JNK) which are members of the mitogen-activated protein kinase (MAPK) family [56]. Another study found that β-ionone inhibited the proliferation of mammary cancer cells via modulating the MAPK pathway [84].

Wiese et al. showed that proline-rich tyrosine kinase 2 (Pyk2) plays a critical role in the signaling cascades initiated after OR51E2 activation (Figure 1). β-Ionone (via OR51E2) activated Pyk2 in prostate cancer cells, possibly mediated via an increase in intracellular calcium concentration. Pyk2 increases the phosphorylation of p38 MAPK and dephosphorylation of N-myc downstream regulated gene 1 (NDRG1). The latter along with Pyk2 are thought to be responsible for the β-ionone mediated anti-proliferative effect in prostate cancer cells. In addition, this study revealed that β-ionone activating OR51E2 resulted in regulation of various proteins involved in processes such as signaling and ion transport in prostate cancer cells [67].

Jones et al. supported the idea that anti-proliferative effect of β-ionone is AR independent [74]. However, a recent study by Xie et al. revealed that the presence of AR is essential for the β-ionone activation of OR51E2. OR51E2 stimulation by β-ionone induced calcium influx in prostate cancer cell lines (LNCaP, C4-2 and DU145). However, it only inhibited prostate cancer proliferation in LNCaP and C4-2 cells, which are expressing AR but not in DU145 cells, which do not express AR. Notably β-ionone-induced activation of OR51E2 inhibited AR nuclear translocation via p38 and JNK-mediated AR phosphorylation (Figure 1). The suppressed AR translocation caused a reduction in AR transactivation and thus retardation of prostate cancer cell growth. This was further supported by in vivo studies that showed that β-ionone suppressed tumor growth in mice and caused an increase in the phosphorylation in AR, p38, and JNK [50].

However, Sanz reported that β-ionone induced prostate cancer (LNCaP) cell invasiveness via activation of phosphatidylinositol-4,5-bisphosphate 3-kinase-ɣ (PI3Kɣ) mediated by OR51E2 stimulation (Figure 1). The βɣ subunit of G protein was responsible to activate PI3Kɣ [65]. A study from Cao et al. showed that β-ionone mediated activation of OR51E2 inhibited the proliferation of C4-2 cells but promoted their invasion consistent with reported data from LNCaP cells [66]. Notably, β-ionone administered to mice with prostate tumors also induced cell metastasis [65,70]. On the other hand, another study found that β-ionone combined with sorafenib (a multi kinase inhibitor) exhibit a synergistic inhibition of metastasis of human hepatoma cells [85]. This was mediated via β-ionone-induced upregulation of the expression of tissue inhibitor matrix metalloproteinase-1 and -2 [85,86], which is a well-accepted approach for metastasis inhibition [85].

Studies showed that β-ionone also inhibited the proliferation in melanocytes via activation of OR51E2 [49]. However, β-ionone decreased the LPA-induced migration and retarded the growth of vertical-growth phase (VGP) melanoma cells by induction of apoptosis [68]. On the other hand, β-ionone increased the melanin level in melanocytes and the percentage of cells with more than two dendrites. Melanogenesis is found to be mediated by β-ionone activation of OR51E2 resulting in activation of adenylate cyclase and thus increase in cAMP and activation of protein kinase A (PKA). PKA might be responsible to upregulate the melanogenic enzyme, tyrosinase (Figure 1). Moreover, activated MAPK (p44/42 and p38) have been reported which could also be the likely regulator of melanogenesis. It was shown that the surface OR51E2 and not cytosolic OR51E2 receive the external signal. On this basis, OR51E2 are assumed to be inserted into the membrane with repetitive β-ionone stimulation of melanocytes [49].

On that note, studies investigated the mechanism of calcium increase in prostate cancer cells upon OR51E2 stimulation. It was found that transient receptor potential vanilloid type 6 (TRPV6) channels are activated downstream of β-ionone-mediated activation of OR51E2. Src kinases play a critical role in the β-ionone mediated influx of calcium; OR51E2 directly activates Src kinase independently of G protein activation (Figure 1) [64].

β-Ionone also caused an increase in intracellular calcium concentration in melanocytes and cultured cells derived from metastatic and VGP as reported in prostate tumor cells [49,68]. This process is also mediated via the activation of surface OR51E2. Calcium from the extracellular space and intracellular stores account for the elevation of calcium levels however, the former was found to be the main significant contributor (Figure 1). Extracellular calcium is influxed via transient receptor potential channels specifically TRPM and not TRPV as reported in prostate cancer cells [49].

Another study showed that β-ionone induced an increase in intracellular calcium in retinal pigment epithelial cells; calcium originated from extracellular space. The increase in calcium was mediated via activation of the cAMP pathway. Even though no channel was identified, L-type calcium or TRP channels (as in prostate and melanocytes) were suggested to be involved [37].

It appears that exogenous β-ionone, provided via diet and fragrance, might activate OR51E2 found in the plasma membrane. However, it is proposed that β-ionone, endogenously produced from BCO2 in the mitochondria, might activate intracellular OR51E2 [37]. Intracellular localization of OR51E2 has been presented in the aforementioned cells [37,49,71]. Based on β-ionone hydrophobic properties that allows it to traverse cellular membrane [49], it appears that exogenous, endogenous β-ionone might activate plasma membrane, or cytosol localized OR51E2.

#### 4.1.2. Cell Cycle Regulatory Protein-Mediated Effects

β-Ionone has been shown to inhibit the proliferation of breast cancer [3,72,73], prostate cancer [74], human colon cancer [75], human gastric adenocarcinoma [79], murine B16 melanoma cells, human leukemia and human colon adenocarcinoma cell lines [3] and in vivo in mammary cancerous glands [76] via induction of cell cycle arrest.

β-ionone has been shown to suppress cell proliferation via arresting the cell cycle at the G1/G0 phase in breast cancer cell line [73], G1 phase in human colon cancer [75] and prostate cancer cell line (DU145, PC-3 cells) [74] and G1/G0 and G2/M phases in human gastric cancer and breast cancer cell lines [72,79].

**Figure 2:**
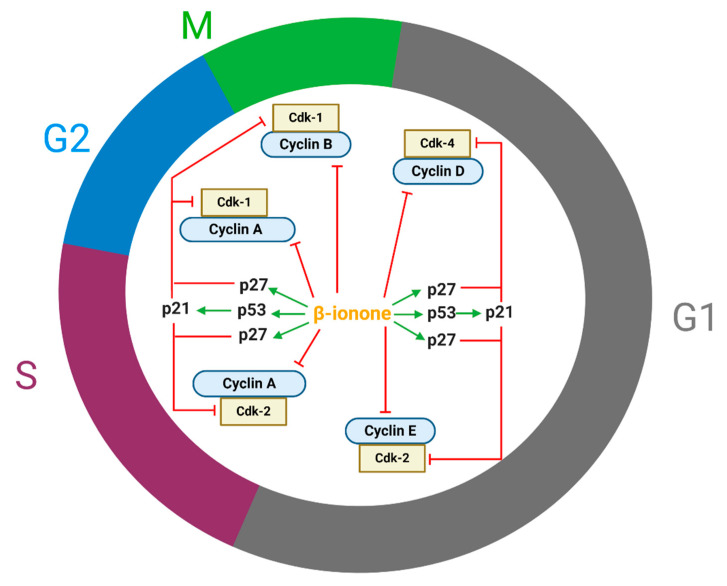
β-Ionone modulates several cell cycle regulatory proteins causing cell cycle arrest in various cancer cells (Cdk-1, cyclin-dependent kinase-1; Cdk-2, cyclin-dependent kinase-2; Cdk-4, cyclin-dependent kinase-4; p27, cyclin-dependent kinase inhibitor; p21, cyclin-dependent kinase inhibitor).

Cell proliferation is regulated by the cell cycle [79]. Cyclin, cyclin dependent-kinases (CDK) and CDK inhibitors (CdkI) play critical role in modulating the cell cycle. Cell cycle arrest was explained (Figure 2) by a reduction in cyclin D1 [72,73,74,76,79], cyclin E, cyclin B1, cyclin A, Cdk 2, [72], Cdk4, [72,73,74,79]. Cdk phosphorylate proteins involved in cell cycle progression. Cdk become activated only when complexed with cyclins to form Cdk-cyclin complexes. Cdk4-Cyclin D, Cdk2-cyclin E, Cdk2-cyclin A, Cdk1-cyclin A and Cdk1-cyclin B regulate early G1 phase, late G1 phase, progression through S phase, G2/entry of M phase and progression through M phase, respectively [87] (Figure 2).

Moreover, β-ionone augmentation in the expression of p27 was reported that could also explain the cell cycle arrest and thus cell growth retardation [79]. It is believed that β-ionone targets p53 in persistent preneoplastic lesions at early stages of heptocarcinognesis [88]. P53 regulate cell cycle arrest primarily via transcriptional activation of p21 [89]. P21 and p27 are CdkI that bind to and block Cdk-cyclin complex activity specifically complex involving cyclin A, B, D and E (Figure 2) [90]. In addition, it is proposed that cell cycle arrest may be modulated via MAPK pathway; as Dong et al. showed that β-ionone inhibited the expression of extracellular signal-regulated kinase and induced the expression of p38 and SAPK/JNK [79].

#### 4.1.3. Apoptotic Effect

**Figure 3:**
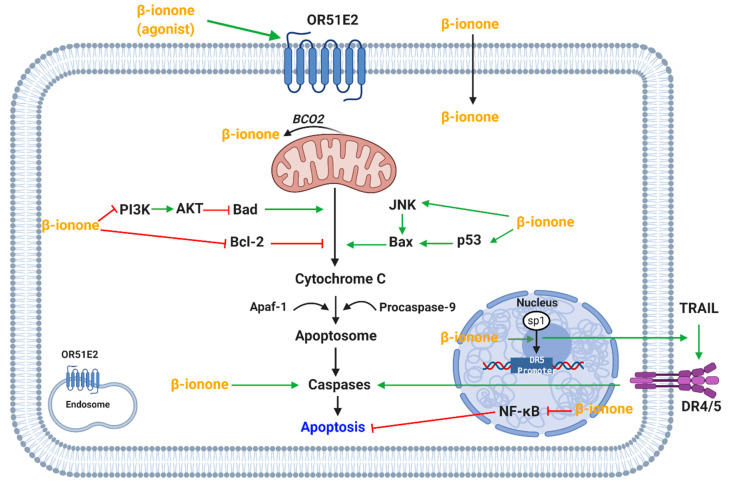
A proposed mechanism of β-ionone modulating pro-apoptotic and anti-apoptotic pathways. (OR51E2, olfactory receptor 51E2; BCO2, β-carotene oxygenase 2; JNK, Jun amino-terminal kinases; AKT, protein kinase B; PI3K, phosphoinositide 3-kinase; Apaf-1, apoptotic protease activating factor 1; TRAIL, tumor necrosis factor related apoptosis-inducing ligand; DR4/5, death receptor 4/5; NF-кB, nuclear factor-κB; transcription factor SP1; specificity protein 1; DR5 promoter, death receptor 5 promoter).

β-Ionone induces apoptosis in mammary cancer glands [76] and breast cancer [73], leukemia [3] and human gastric adenocarcinoma cell lines [9,77,78,79] via induction of apoptosis. β-Ionone-induced apoptosis was mediated via reducing the expression of anti-apoptotic protein, Bcl-2, and increasing the expression of pro-apoptotic protein, Bax (Figure 3) [9,76,79]. In addition, β-ionone increased the expression of cleaved caspase-3 in breast cancer and human gastric adenocarcinoma cell lines [9,73,78,79]. It is believed that β-ionone targets p53 in persistent preneoplastic lesions at early stages of hepatocarcinogenesis (Figure 3) [88]. However, the β-ionone induction of apoptosis was previously shown to be independent of mutated p53 function [3].

In addition, reduction in the level of phosphorylated PI3K (phosphoinositide 3-kinases) proteins and AKT expression has been reported. The PI3K-AKT pathway could also explain the apoptosis of cells as the pathway is involved in cell proliferation, survival and cell signaling (Figure 3) [9]. Further investigations supported the role of MAPK pathway in cell proliferation and apoptosis as an increase in phosphorylated-p38 was documented [73]. These studies reveal that apoptosis might be mediated via MAPK or PI3K-AKT pathway.

Liu et al. found that β-ionone caused a dose-dependent inhibition of mammary carcinogenesis in rats; tumor multiplicity decreased and time to tumor appearance time increased. Furthermore, a decrease in proliferating cell nuclear antigen and an increase in nuclear fragmentation was found [76]. Additionally, β-ionone was found to sensitize the cells to TRAIL (Tumor necrosis factor related apoptosis-inducing ligand)-stimulated apoptosis. This is mediated via increasing the direct binding of transcription factor Sp1 to the DR5 promoter site, abolishing NF-κB activation and decreasing the expression of anti-apoptotic proteins (Figure 3) [91] Another study found that β-ionone targets NF-кB in persistent preneoplastic lesions at early stages of hepatocarcinogenesis [88].

Janakiram et al. found that β-ionone induces apoptosis possibly via acting as a ligand at the retinoid X receptor-α (RXR-α) in human colon cancer cells. β-ionone was found to increase the levels of expression of RXR-α mRNA dose dependently in cells. In addition, RXR-α expression is found to be lessened in other cancer cells [75]. Notably, the expression of RXR-α mRNA was decreased not only in rat colonic adenocarcinoma but also in basal cells in prostate cancer; proposing its influence in cancer progression [75,92]. This is further supported by the structure similarity depicted between β-ionone and β-carotene/vitamin A, recognized to bind to RXRs [75]. However, β-ionone in vivo effect on rat colonic aberrant crypt focci was controversial as studies reported inhibition or no effect on aberrant crypt focci [75,93].

#### 4.1.4. HMG CoA Reductase-Mediated Effect

3-Hydroxy-3-methylglutaryl coenzyme A (HMG CoA) reductase is responsible for the synthesis of mevalonate [3]. Mevalonate is a precursor of sterol molecules such as cholesterol and nonsterol isoprenoids such as dolichol, ubiquinone, farnesyl pyrophosphate and geranylgeranyl pyrophosphate [94]. While cholesterol plays an important role in membrane integrity, nonsterol isoprenoids are involved in various cellular processes. The geranylgeranyl pyrophosphate and farnesyl pyrophosphate prenylate proteins (post-translational modification) such as Ras, Rho and Rac GTPase family [95,96], which in turn play vital role in cell proliferation, angiogenesis and survival [97,98].

**Figure 4:**
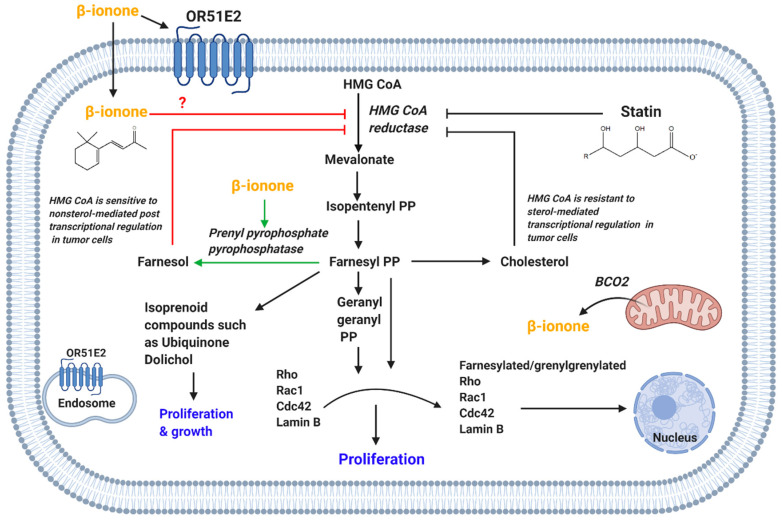
β-Ionone-mediated inhibition of HMG-CoA reductase resulting in anti-proliferative effect and reduction in the level of cholesterol. (OR51E2, olfactory receptor 51E2; BCO2, β-carotene oxygenase-2; HMG CoA, 3-hydroxy-3-methylglutaryl coenzyme A; PP, pyrophosphate).

The inhibition of HMG CoA reductase has been associated with cell apoptosis, cell cycle arrest and proliferation suppression [99,100,101]. Statins, which are well-established inhibitors of HMG-CoA reductase, are reported to possess anticancer activity. In tumor cells, the HMG-CoA reductase is resistant to sterol-regulated feedback inhibition (Figure 4) [8,102] and thus its upregulated in various types of cancers [103,104,105,106,107]. However, HMG CoA reductase in tumor cells is sensitive to posttranscriptional inhibition mediated by nonsterol compounds. Isoprenoids were also found to post transcriptionally suppress HMG-CoA reductase activity [100,108].

Dietary β-ionone suppressed serum total cholesterol level and HMG CoA activity in chickens, while increasing the HDL level [80,81]. β-Ionone-induction of chemoprevention in rat mammary carcinogenesis [76,109,110] was parallel to its inhibition of HMG-CoA reductase [109]. Espindola et al. reported that the higher doses of β-ionone (16 mg/100 g) inhibited cell proliferation of preneoplastic lesions and caused smaller visible hepatocyte nodules in initial phases of hepatocarcinogenesis. Notably, the level of total plasma cholesterol decreased with administration of higher doses of β-ionone [82]. Other studies found that β-ionone suppression of the growth of Murine B16 melanoma, breast cancer, human leukemia, human colon adenocarcinoma [3] and prostate tumor cell lines was conveyed via reducing HMG CoA reductase activity [74]. This was further supported by the synergism anti-proliferative activity reported between β-ionone and either lovastatin or trans, trans-farnesol, compounds involved in reductase suppression [3,74].

β-Ionone suppression of HMG-CoA activity is mediated via posttranscriptional action. The β-ionone inhibition of HMG CoA activity was explained by indirect facilitation of HMG CoA degradation. This is mediated via induction in the activity of prenyl pyrophosphate pyrophosphatase [81], which will in turn increase the level of farnesol, a nonsterol mevalonate product that degrades HMG CoA reductase (Figure 4) [111]. Moreover, β-ionone and lovastatin affected the post-translational modification of lamin B, which is an important action for the assembly of nuclear membrane during interphase. Thus, it is suggested that β-ionone mediated inhibition of HMG CoA-reductase inhibits lamin B nuclear localization and thus causes the DNA to be vulnerable to endonucleases. This action will result in apoptosis as depicted with β-ionone and lovastatin. Moreover, β-ionone and lovastatin caused cell cycle arrest at G1 phase [3]. Therefore, the β-ionone and statin shared HMG-CoA regulation and synergistic anti-proliferative actions support β-ionone usage as adjunct cancer therapy to reduce statins’ adverse effects [96].

We suggest that β-ionone might be directly inhibiting HMG-CoA reductase in a similar manner to statin, as β-ionone looks like it is sharing a similar backbone to statin pharmacophore (Figure 4) [112]. In addition, a recent study showed that compounds that are completely distinct from statin structure were able to inhibit HMG-CoA reductase [113].

Nevertheless, Duncan and his colleagues showed that β-ionone inhibit breast cancer cells’ proliferation and cell cycle without affecting HMG CoA reductase activity [72]. β-Ionone was found to have a chemoprotective effect as it decreased the mean number of visible hepatocyte nodules [82,114], and persistent preneoplastic lesions [88,114] and caused DNA damage throughout the initiation of hepatocarcinogenesis in rats. Notably, β-ionone increased the hepatic HMG-CoA reductase mRNA levels but decreased the level of total plasma cholesterol in rats with hepatic cancer. This increase in the mRNA levels of the reductase could be a compensatory mechanism subsequent to HMG CoA degradation [114]. Farnesol had similar effects on HMG CoA reductase and cholesterol [115], which supports the documented β-ionone modulation of farnesol activity.

#### 4.1.5. Antioxidant-Mediated Effect

**Figure 5:**
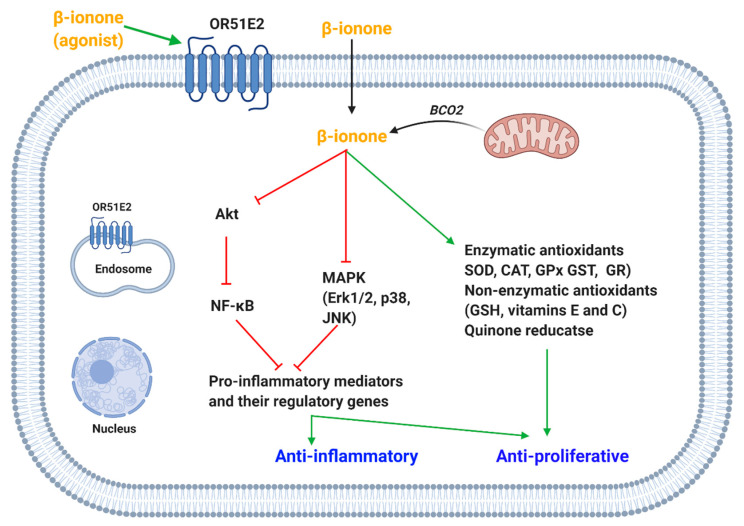
β-Ionone inhibition of pro-inflammatory mediators and induction/restoration of antioxidants causing anti-proliferative and anti-inflammatory effects. (OR51E2, olfactory receptor 51E2; BCO2, β-carotene oxygenase-2; MAPK, mitogen-activated protein kinase; JNK, Jun amino-terminal kinases; AKT, protein kinase B; NF-кB, nuclear factor-κB; Erk1/2, extracellular signal-regulated kinase 1/2; SOD, superoxide dismutase; CAT, catalase; GPx, glutathione peroxidase; GST, glutathione-S-transferase; GR, glutathione reductase; GSH, glutathione).

Asokkumar proposed that β-ionone anti-proliferative activity is attributable to its antioxidant properties. Benzo(a)pyrene, a well-known carcinogen, was chosen to induce lung cancer in mice as it is believed to generate reactive oxygen species and initiate proliferative changes. The anti-proliferative effect of β-ionone was shown via a decrease in proliferating cell nuclear antigen expression and restoration of normal levels of cancer marker enzymes (carcinoembryonic antigen and neuron-specific enolase). β-ionone administered to mice restored the activities of enzymatic (such as catalase, glutathione peroxidase (GPx), glutathione-S-transferase, glutathione reductase (GR) and superoxide dismutase (SOD)) and non-enzymatic antioxidants (such as glutathione (GSH), vitamins E and C) to levels detected prior benzo(a)pyrene treatment (Figure 5) [116].

Another study showed that β-ionone had chemoprotective effects and suppressed the 7,12-dimethylbenz(a)anthracene-induced mammary carcinogenesis in rats. It increased the activities of antioxidant enzymes (GPx, GR, SOD) and GSH. The antioxidant effect of β-ionone was further supported by a reduction in lipid peroxidation, malondialdehyde (marker for oxidative stress) and nitric oxide (a reactive nitrogen species) [110,116]. Dong et al. showed that β-ionone application on Hepa1c1c7 cells augmented the quinone reductase activity, which is a phase II detoxifcation enzyme [73]. The exact mechanism of how β-ionone imparts an antioxidant effect is still not clear. However, the effect on quinone reductase might be explained via modulation of p38, which is a negative modulator of the activation of phase II detoxifying enzymes [117].

#### 4.1.6. Pro-Inflammatory Molecules-Mediated Effects

β-Ionone inhibits COX-2 expression in human gastric cancer cells and rat mammary tumor tissues, which might explain its anti-proliferative effect. Moreover, the release of prostaglandin E2 (PGE_2_) in human gastric cancer cells was reduced [73]. Kang supported the β-ionone inhibitory activity on the expression of lipopolysaccharide (LPS)-induced pro-inflammatory mediators. It was reported that β-ionone attenuated the release of nitric oxide (NO), prostaglandin E2 (PGE2) and tumor necrosis factor-α (TNF-α) and the protein and mRNA expression of inducible NO synthesis (iNOS), cyclooxygenase-2 and TNF-α in LPS-induced BV2 microglia cells. It was found that β-ionone regulates these inflammatory mediators via inhibition of NF-κB and MAPK pathway (Figure 5). The authors showed that β-ionone decreased the DNA-binding activity of NF-κB by retardation of Akt activity. In addition, β-ionone inhibited the phosphorylation of MAPKs (Erk, p38 and JNK) which are important regulators of the release of pro-inflammatory mediators [83].

#### 4.1.7. Antimicrobial Effects

Griffin and his colleagues studied the role of structural groups in determining the antimicrobial activity of several terpenoids. β-ionone inhibitory activity was found against *Escherichia coli* and *Candida albicans* [118]. β-Ionone inhibited the growth of *Aspergillus flavus* and sporulation of both *A. flavus* and *A. parasiticus*. Furthermore, the morphology of asexual reproductive structures were altered upon β-ionone exposure. Notably, the aflatoxin accumulation was dramatically reduced in spores of *A. parasiticus* [119].

### 4.2. α-Ionone

As previously discussed, β-ionone acts as an agonist of OR51E2. On the other hand, α-ionone activity on OR51E2 is not as clear. Several studies reported that α-ionone antagonize OR51E2 which prevented or suppressed the effects of β-ionone, notably it has been utilized to confirm OR51E2 activation by β-ionones [45,49,56,65]. For example, Neuhaus found that α-ionone had no effect on the intracellular calcium concentration and it inhibited the β-ionone induced increase in intracellular calcium and anti-proliferative effect in prostate cancer cells [56].

In contrast, Sanz et al. showed that α-ionone is a real agonist of OR51E2. Notably, α-ionone primarily induced growth of LNCaP cells and prostate tumors in mice whereas β-ionone increased cell invasiveness. These findings are in agreement with biased agonism phenomena reported with GPCR [120]. Thus, it is proposed that each ionone induces a distinct downstream signaling cascade depending on the G protein or β-arrestin coupled [70].

In addition, α-ionone elicited distinct responses depending on its dose; moderate doses augmented LNCaP cell invasiveness while higher doses did not (instead it sustained cell growth). It is proposed that α-ionone modulates different cellular signaling cascades depending on doses. For instance, ionone might be partially or fully activating OR51E2 that triggers distinct downstream signaling cascades. It could also mean that α-ionone is antagonizing OR51E2 at higher doses. Nevertheless, the higher doses of α-ionone activating OR51E2 in transfected HEK293 cells supported the former hypothesis [70].

### 4.3. Ionone Derivatives

Several compounds have been synthesized and shown to exert anticancer, anti-inflammatory and antimicrobial activity.

#### 4.3.1. Anti-Cancer

**Figure 6:**
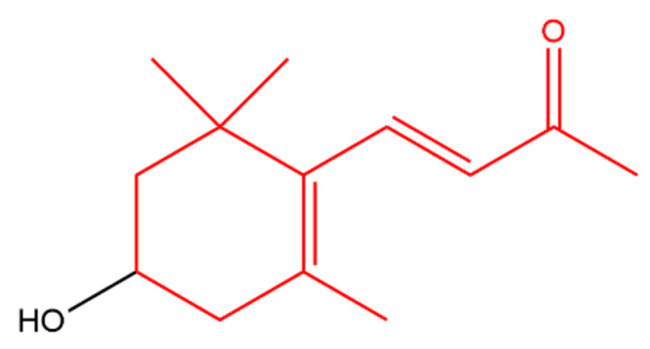
3-Hydroxy-β-ionone. The β-ionone moiety is shown in red.

3-Hydroxy-β-ionone (Figure 6) retarded the proliferation, colony formation and cell migration of squamous cell carcinoma. In addition, it caused apoptosis and cell cycle arrest at G2/M phase in these cells. Apoptosis was indicated by an increase in cleaved caspases and Bax with a decrease in Bcl-2 (as seen with β-ionone, Figure 3) [121]. It appears that 3-hydroxy-β-ionone is produced endogenously from BCO2 asymmetric cleavage of zeaxanthin, a carotenoid with a 3-hydroxy-β-ionone ring [40].

**Figure 7:**
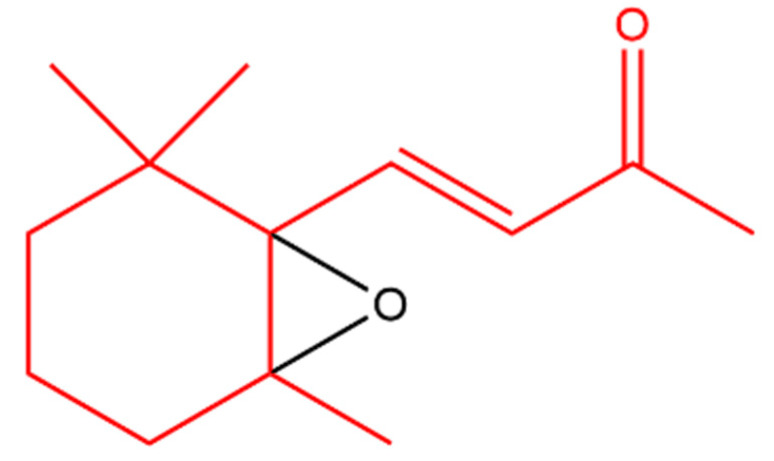
5,6-Epoxy-β-ionone. The β-ionone moiety is shown in red.

β-Ionone was also found to antagonize 12-O-tetradecanoylphorbol-13-acetate (TPA); a tumor-inducing compound. Another derivative, 5,6-epoxy-β-ionone (Figure 7), displayed more pronounced TPA inhibition than β-ionone in bovine lymphocytes [122].

**Figure 8:**
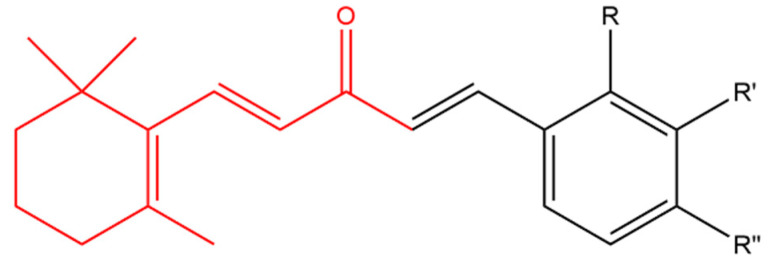
(Compound **a**). R = H, R’ = H, R’’ = NO_2_; [(1*E*,4*E*)-1-(4-nitrophenyl)-5-(2,6,6-trimethylcyclohex-1-en-1-yl) penta-1,4-dien-3-one]; (compound **b**). R = H, R’ = CF_3_, R’’ = H; [(1*E*,4*E*)-1-(3-(trifluoromethyl)phenyl)-5-(2,6,6-trimethylcyclohex-1-en-1-yl)penta-1,4-dien-3-one] (compound **c**). R = NO_2_, R’ = H, R” = H [(1*E*,4*E*)-1-(2-nitrophenyl)-5-(2,6,6-trimethylcyclohex-1-en-1-yl)penta-1,4-dien-3-one]; (compound **d**). R = Cl, R’ = H, R” = H [(1*E*,4*E*)-1-(2-chlorophenyl)-5-(2,6,6-trimethylcyclohex-1-en-1-yl)penta-1,4-dien-3-one]; (compound **e**). R = Br, R’ = H, R” = H; [(1*E*,4*E*)-1-(2-bromophenyl)-5-(2,6,6-trimethylcyclohex-1-en-1-yl)penta-1,4-dien-3-one]. The β-ionone moiety is shown in red.

Sharma and his team combined β-ionone with various aldehydes to prepare a series of chalcones. Chalcones are plant-derived ketones composed of two aromatic rings that possess anticancer and antimicrobial activity. All derivatives inhibited the proliferation of various cancer cell lines including prostate, breast, CNS, cervix and liver. Compound (a) in Figure 8 was more effective in inhibiting cell proliferation compared to other derivatives. It was noticed that compounds having electron-withdrawing group on the *para* position of the ring moiety were more cytotoxic than compounds with electron-donating group in the *ortho* position. Further studies using Chinese hamster ovary cells, showed that compound (a) caused cytotoxicity via induction of apoptosis and cell cycle arrest at G0 phase [123].

Zhou et al. designed a variety of β-ionone-based chalcones; they exhibited various level of cytotoxic effect on a variety of prostate cancer cell lines, including LNCaP, MDA-PCa-2b, 22Rv1, C4-2B and PC-3. It is believed that compounds with an electron withdrawing group at the meta position of the ring moiety are more potent than compound with an electron donating group at the para position. Compound (b) in Figure 8 was found to induce the strongest cytotoxic effect in androgen dependent and independent prostate cancer cells even in comparison to β-ionone in the context of LNCaP and PC-3 cells. Furthermore, compound (b) was found to elicit anti-androgenic activity; as it inhibited the DHT-induced transactivation of wild type AR and various mutated ARs (as seen with β-ionone, Figure 1) [124].

**Figure 9:**
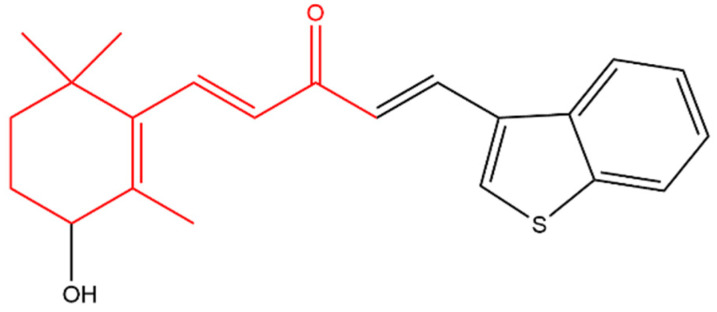
(1*E*,4*E*)-1-(Benzo[b]thiophen-3-yl)-5-(3-hydroxy-2,6,6-trimethylcyclohex-1-en-1-yl)penta-1,4-dien -3-one. The β-ionone moiety is shown in red.

**Figure 10:**
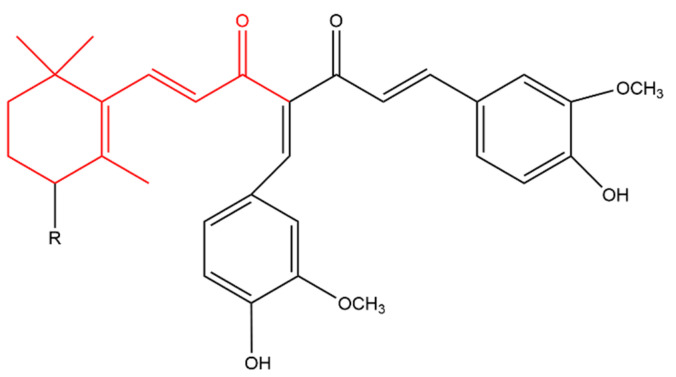
(Compound **a**). R = H; [(1*E*,6*E*)-4-((*Z*)-4-hydroxy-3-methoxybenzylidene)-1-(4-hydroxy-3-methoxy phenyl)-7-(2,6,6-trimethylcyclohex-1-en-1-yl)hepta-1,6-diene-3,5-dione] (compound **b**). R = OH; [(1*E*,6*E*)-1-(3- hydroxy-2,6,6-trimethylcyclohex-1-en-1-yl)-4-((*Z*)-4-hydroxy-3-methoxybenzylidene)-7-(4-hydroxy-3-methoxyphenyl)hepta-1,6-diene-3,5-dione]. The β-ionone moiety is shown in red.

Zhou later synthesized a variety of β-ionone-based compounds; the compound shown in Figure 9 and compound (a) in Figure 10 were the most potent inhibitors of the proliferation of various prostate cancer cells (including LNCaP, MDA-PCa-2b, C4-2B and 22Rv1). In addition, these compounds acted as full antagonists of the wild type AR and a variety of clinically important mutated ARs in PC-3 cells [125,126]. Molecular modeling revealed that the two bulky side-chains of compound (a) in Figure 10 are critical for mutated AR antagonism. On the other hand, compound (a) elicited cytotoxic activity against PC-3 cells, which lack AR, suggesting that compound is not only targeting AR [125]. Further investigations revealed that the compounds in Figure 9 and Figure 10 inhibited NF-KB activation and interleukin-6 (IL-6) release. Compound (b) in Figure 10, a derivatives of the compound in Figure 9, also suppressed prostate tumor cell proliferation (C4-2B, 22Rv1, PC-3 and DU-145 cells), AR and NF-κB signaling. In addition, compound (b) resulted in apoptosis as it caused caspase 3 activation in LNCaP cells [127].

#### 4.3.2. Anti-Inflammatory

**Figure 11:**
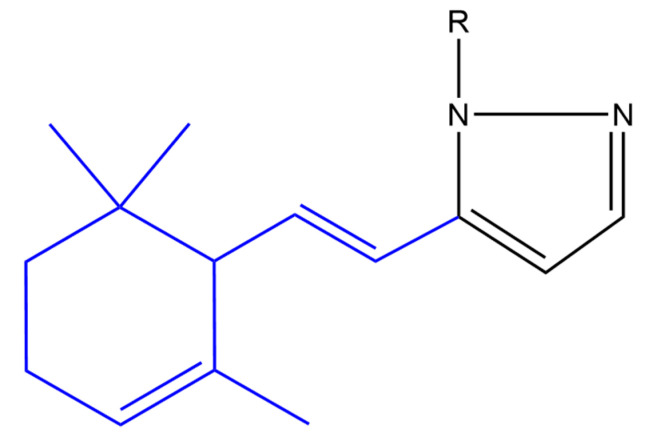
(Compound **a**). R = H; [(*E*)-5-(2-(2,6,6-trimethylcyclohex-2-en-1-yl)vinyl)-1H-pyrazole] (compound **b**). R = 4-amino-4*H*-1,2,4-triazole-3-thiol; [(E)-4-amino-5-(5-(2-(2,6,6-trimethylcyclohex-2-en-1-yl)vinyl)-1*H*-pyrazol-1 -yl)-4*H*-1,2,4-triazole-3-thiol] (compound **c**). R = phenyl [(*E*)-1-phenyl-5-(2-(2,6,6-trimethylcyclohex-2-en-1-yl)vinyl)-1*H*-pyrazole]. The α-ionone moiety is shown in blue.

Balbi and co-workers synthesized heterocyclic ionone-like derivatives. However, they focused on α-ionone derivatives, as they were easier to synthesize. Almost half of the synthesized compounds inhibited superoxide anion production in neutrophils in a dose-dependent manner, in which compounds (a) and (b) (Figure 11) were found to be most active. These two compounds also inhibited the chemotactic response of neutrophils in a dose dependent manner. It was found that these derivatives inhibited neutrophil chemotaxis at lower concentrations compared to nonsteroidal anti-inflammatory drugs. It was noticed that a pyrazole ring and its substitution at position 1 seems to be very important for activity whereas an unsubstituted pyrazole (compound (a)) or an aminotriazole (compound (b)) substituent elicited the highest activity [128].

In addition, a collection of β-ionone-derived curcumin analogs inhibited the secretion of pro-inflammatory cytokines including TNF-α and IL-6, following LPS stimulation in a dose dependent manner. These analogs were found to be more potent than curcumin. The most active compound (c) (Figure 8) inhibited LPS-induced septic death in mice. The study showed that having any substituent at the second position of the phenyl group resulted in normal activity against inflammatory cells while a substituent at fourth position of the phenyl group reduced the activity of the compounds. In addition, it showed that having chloro group substituents at the third and fourth position of the phenyl group displayed normal activity against inflammatory cells however replacing the chloro groups with methyl groups almost abolished this activity [129].

#### 4.3.3. Antimicrobial Effect

**Figure 12:**
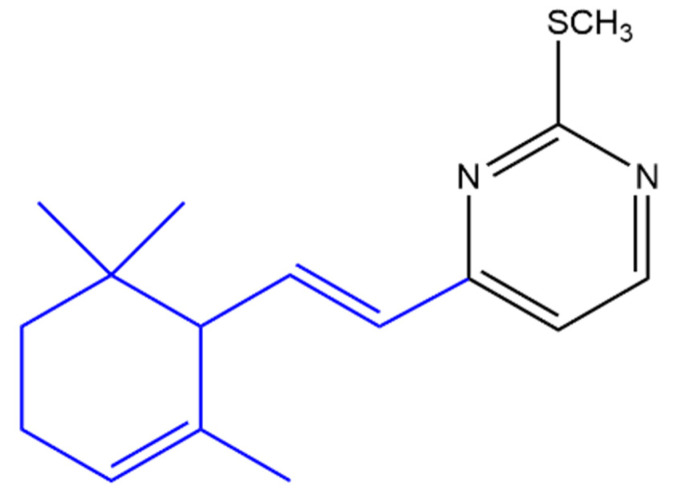
(*E*)-2-(Methylthio)-4-(2-(2,6,6-trimethylcyclohex-2-en-1-yl)vinyl)pyrimidine. The α-ionone moiety is shown in blue.

A variety of heterocyclic ionone-like derivatives displayed moderate to good activity against *C. albicans*. Only compound (c) (Figure 11) and the compound shown in Figure 12 exhibited good activity against *Pseudomonas aeruginosa*, *Propionibacterium acnes* and *E. coli*. Most of the compounds showed good activity against *Staphylococcus aureus*. The addition of a phenyl group at the position-1 of the pyrazole ring is important for the antibacterial activity however, the substitution with 2,4-dinitrophenyl group in the same position reduced the inhibitory activity against *E. coli*, *P. aeruginosa* and *S. aureus*. It was concluded that lipophilicity is an important factor in increasing the activity against broader spectrum of microbial species [130].

Sharma and his team combined β-ionone with various aldehydes to prepare a series of chalcones. The active compounds exhibited promising antimicrobial activity against a variety of Gram negative bacteria *(E. coli*, *P. aeruginosa, Salmonella typhimurium),* Gram positive bacteria (*S. aureus*, *Bacillus subtilis*) and fungal strains (*A. niger*, *Saccharomyces cerevisiae*, *C. albicans*). In addition, further testing showed that some of the active compounds were also active against methicillin resistant *S. aureus*. Compound (d) in Figure 8 displayed the highest anti-bacterial activity against both Gram negative and positive bacteria while compound (e) showed the uppermost antifungal activity against species including *A. niger* and *S. cerevisiae*. It was noticed that compounds with electron withdrawing substituents on the aromatic ring mostly displayed better antibacterial activity in comparison to compounds with electron releasing groups [131].

**Figure 13:**
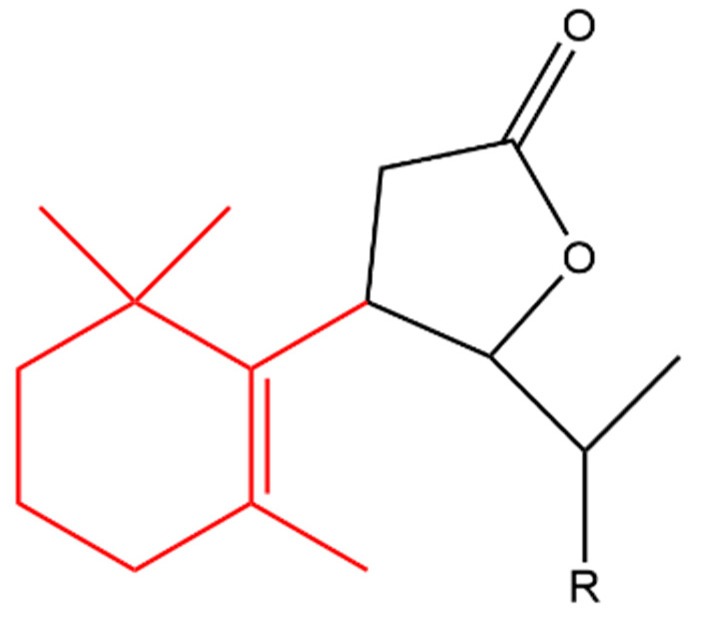
(Compound **a**). R = Cl; [10-(11-chloroethyl)-7-(1,1,5-trimethylcyclohex-5-en-6-yl)dihydrofuran-2(3H)-one]; (compound **b**). R = OH [10-(11-hydroxyethyl)-7-(1,1,5-trimethylcyclohex-5-en-6-yl)dihydrofuran-2(3H)-one]; (compound **c**). R = Br [10-(11-bromoethyl)-7-(1,1,5-trimethylcyclohex-5-en-6-yl)dihydrofuran-2(3H)-one]. The β-ionone moiety is shown in red.

β-Ionone was also used as a starting material to produce a variety of synthetic halolactones and microbiologically obtained hydroxylactones. All of the derivatives inhibited the growth of *B. subtilis*, *S. aureus* and *E.coli*, however, the chlorolactone (compound (a) in Figure 13) and hydroxylactone (compound (b)) completely inhibited the growth of *B. subtilis* and *S. aureus*, respectively. In addition, all of the compounds increased the growth of *A. niger* and *Fusarium linii* except the bromolactone (compound (c) in Figure 13) which completely inhibited the growth of *A. niger* [132].

**Figure 14:**
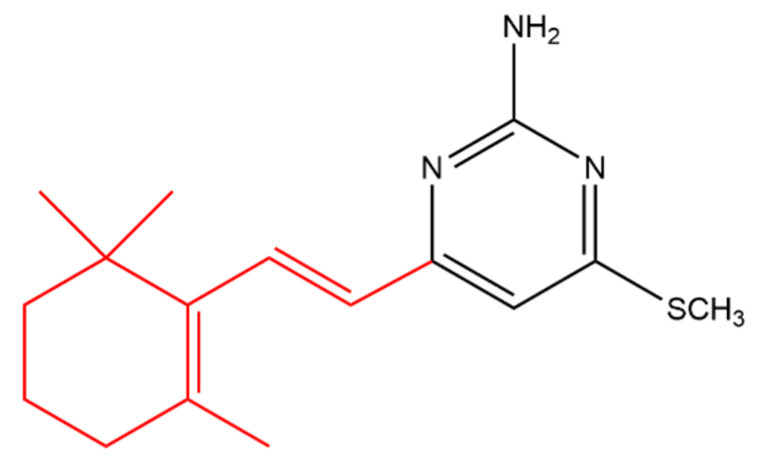
(*E*)-4-(methylthio)-6-(2-(2,6,6-trimethylcyclohex-1-en-1-yl)vinyl)pyrimidin-2-amine. The β-ionone moiety is shown in red.

Suryawanshi and his team synthesized several terpenyl pyrimidines substituted with 4-N in which the starting material (ketene dithioacetal) was made available from β-ionone. The 4-thiomethoxy substituted pyrimidine (Figure 14) caused 66% in vivo inhibition of Leishmania donovani in hamsters. It was noticed that the 4-thiomethoxy group is very important for the anti-leishmanial activity as its replacement with various primary and secondary amines resulted in activity loss [133].

## 5. Conclusions and Outlook

Ionone, specifically β-ionone, can either be endogenously produced via BCO2 or exogenously supplied via the diet. β-Ionone, whether of an endogenous or exogenous origin, possess anticancer, chemopreventive, cancer promoting, melanogenesis, anti-inflammatory and antimicrobial activity. β-Ionone mediates these effects via activation of OR51E2 and modulation of HMG CoA reductase, cell cycle regulatory proteins, pro- and anti- apoptotic pathways, pro-inflammatory cytokines and anti-oxidant enzymes. The activation of OR51E2 causes regulation of the activity of various kinases and an increase in intracellular calcium. OR51E2 activation results in anti-proliferative effect and metastasis in prostate cancer cells, anti-proliferative effect and melanogenesis in melanocytes and proliferation and metastasis in retinal pigment epithelial cells.

The wide spectrum of effects and various signaling cascades attributed to β-ionone might be due to the different cell repertoire [37]. In addition, β-ionone might be interacting with a wide range of receptors, not constrained to OR51E2, such as the reported interaction with AR and RXR-α. Moreover, β-ionone might be activating cytosolic OR51E2 in various intracellular compartments. This is based on β-ionone hydrophobic properties that allows it to traverse cellular membrane.

β-Ionone and α-ionone derivatives exhibit anti-inflammatory, anti-microbial and anticancer effects. Further studies are recommended to investigate the structure activity relationship between β- and α-ionone derivatives and the various reported physiological effects. However, most of the studies are in agreement that electron-withdrawing substituents on the ring moiety attached to β-ionone yield better anti-cancer and anti-microbial activity.

Overall, the data demonstrates that β-ionone is a promising scaffold for cancer, inflammation and infectious disease research and thus is more than a violet’s fragrance.

## Figures and Tables

**Table 1:** The chemical structure of α-ionone and β-ionone.

	α-Ionone	β-Ionone	Reference
Chemical structure depiction	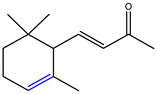	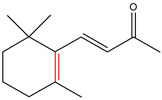	[10,11]

**Table 2:** β-carotene oxygenase 1 (BCO1) and β-carotene oxygenase 2 (BCO2) enzymes have different site and products of β-carotene cleavage, substrate specificity, cellular compartmentalization and human tissue expression.

	β-Carotene Oxygenase 1 (BCO1)	β-Carotene Oxygenase 2 (BCO2)
**Site of Cleavage**	Symmetric–central	Asymmetric–eccentric
**Products of the cleavage of β-carotene**	Two entities of all-trans retinal	β-ionone and β-apo-10′-carotenal
**Substrate specificity**	Pro-vitamin carotenoids	Pro-vitamin and non-pro-vitamin carotenoids
**Cellular compartmentalization**	Cytoplasm	Inner mitochondrial membrane
**Human tissue expression**	Tissues that are expressing BCO1 were also found to express BCO2 however only BCO2 was detected in endometrial connective tissue, endocrine pancreas and cardiac and skeletal muscle	
